# Involvement of a Novel Variant of *FGFR1* Detected in an Adult Patient with Kallmann Syndrome in Regulation of Gonadal Steroidogenesis

**DOI:** 10.3390/ijms26062713

**Published:** 2025-03-18

**Authors:** Yoshiaki Soejima, Yuki Otsuka, Marina Kawaguchi, Kohei Oguni, Koichiro Yamamoto, Yasuhiro Nakano, Miho Yasuda, Kazuki Tokumasu, Keigo Ueda, Kosei Hasegawa, Nahoko Iwata, Fumio Otsuka

**Affiliations:** 1 Department of General Medicine, Okayama University Graduate School of Medicine, Dentistry and Pharmaceutical Sciences, 2-5-1 Shikata-cho, Kitaku, Okayama 700-8558, Japan; p32v0ja8@s.okayama-u.ac.jp (Y.S.); otsuka@s.okayama-u.ac.jp (Y.O.); p1ka7qnp@s.okayama-u.ac.jp (M.K.); oguni-gim@okayama-u.ac.jp (K.O.); koichiro.yamamoto@okayama-u.ac.jp (K.Y.); y-nakano@okayama-u.ac.jp (Y.N.); pf8n7eyn@s.okayama-u.ac.jp (M.Y.); tokumasu@okayama-u.ac.jp (K.T.); kuedampo@okayama-u.ac.jp (K.U.); nao53mayflower@gmail.com (N.I.); 2 Department of Pediatrics, Okayama University Hospital, 2-5-1 Shikata-cho, Kitaku, Okayama 700-8558, Japan; haseyan@md.okayama-u.ac.jp

**Keywords:** fibroblast growth factor receptor 1 (FGFR1), gynecomastia, Kallmann syndrome (KS), osteoporosis and steroidogenesis

## Abstract

Fibroblast growth factor receptor 1 (FGFR1), also known as KAL2, is a tyrosine kinase receptor, and variants of *FGFR1* have been detected in patients with Kallmann syndrome (KS), which is a congenital developmental disorder characterized by central hypogonadism and anosmia. Herein, we report an adult case of KS with a novel variant of *FGFR1*. A middle-aged male was referred for a compression fracture of a lumbar vertebra. It was shown that he had severe osteoporosis, anosmia, gynecomastia, and a past history of operations for cryptorchidism. Endocrine workup using pituitary and gonadal stimulation tests revealed the presence of both primary and central hypogonadism. Genetic testing revealed a novel variant of *FGFR1* (c.2197_2199dup, p.Met733dup). To identify the pathogenicity of the novel variant and the clinical significance for the gonads, we investigated the effects of the *FGFR1* variant on the downstream signaling of FGFR1 and gonadal steroidogenesis by using human steroidogenic granulosa cells. It was revealed that the transfection of the variant gene significantly impaired FGFR1 signaling, detected through the downregulation of SPRY2, compared with that of the case of the forced expression of wild-type *FGFR1*, and that the existence of the variant gene apparently altered the expression of key steroidogenic factors, including StAR and aromatase, in the gonad. The results suggested that the novel variant of *FGFR1* detected in the patient with KS was linked to the impairment of FGFR1 signaling, as well as the alteration of gonadal steroidogenesis, leading to the pathogenesis of latent primary hypogonadism.

## 1. Introduction

Fibroblast growth factor receptor 1 (FGFR1), also known as KAL2, is a tyrosine kinase receptor for fibroblast growth factor (FGF), and it regulates cell proliferation, differentiation, and migration. *FGFR1*, the gene encoding FGFR1, is expressed mainly in Rathke’s pouch and the ventral diencephalon during the embryonic period. It has been reported that variants of *FGFR1* cause Kallmann syndrome (KS) [1], and variants have been detected in 10% of KS cases [2,3].

KS is a congenital developmental disorder characterized by hypogonadotropic hypogonadism and anosmia that results from the incomplete embryonic migration of gonadotropin-releasing hormone (GnRH)-synthesizing neurons and olfactory neurons [4]. Patients show various symptoms and signs of testosterone (T) deficiency, including incomplete or delayed sexual development, loss of body (ancillary and pubic) hair, testicular atrophy, reduced sexual desire, erectile dysfunction, gynecomastia, eunuchoidal body proportions, infertility, and hot flashes. Of note, low-trauma fracture and osteoporosis are also among the suggestive symptoms and signs of hypogonadism and can be important clues for diagnosing KS [5]. When hypogonadism is confirmed by measuring T levels, primary (testicular) and secondary (hypothalamic or pituitary) hypogonadism are distinguished by measuring luteinizing hormone (LH) and follicle-stimulating hormone (FSH) levels. In men with secondary hypogonadism, clinicians should consider pituitary function tests and pituitary magnetic resonance imaging (MRI) to determine the cause of gonadotropin deficiency, such as idiopathic hypogonadotropic hypogonadism (IHH), which is divided into KS with anosmia and normosmic IHH (nIHH).

KS is often accompanied by cryptorchidism, testicular atrophy, micropenis, orofacial clefts and/or tooth agenesis, renal anomalies, bimanual synkinesis, and hearing loss [6]. KS exhibits different inheritance patterns and genetic heterogeneity, including X-linked-recessive, *ANOS1*; autosomal-recessive, *PROK2* and *PROKR2*; and autosomal-dominant, *FGFR1*, *FGF8*, and *CHD7* [7]. *FGFR1* was the first gene to be identified as an autosomal dominant form of KS [1], and more than 140 *FGFR1* variants have so far been reported [8]. Herein, we report an adult case of KS with a novel variant of *FGFR1*.

## 2. Case Presentation

**Figure 1:**
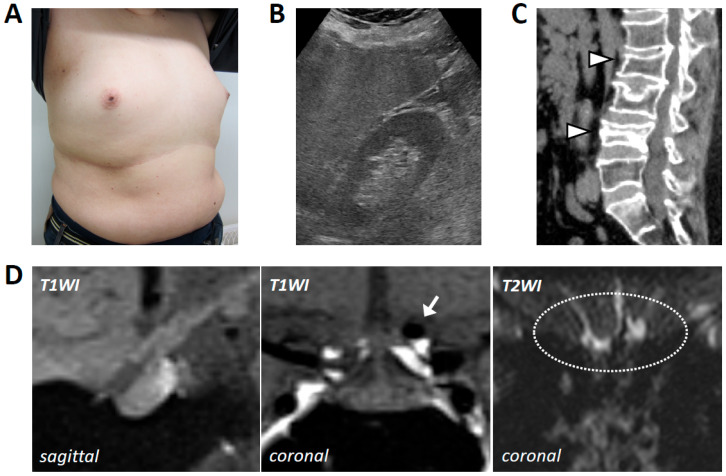
Clinical presentations of the patient with a novel variant of *FGFR1*. (**A**) The patient showed gynecomastia and loss of axillary and pubic hair. (**B**) Abdominal ultrasonography revealed findings of fatty liver. (**C**) Spinal CT showed compression fractures of the second and fourth lumbar vertebrae (shown by arrowheads). (**D**) Sagittal (left), coronal T1-weighted (center), and coronal T2-weighted MRI images of the patient. The MRI revealed no abnormalities in the hypothalamus or pituitary gland, but an internal carotid artery aneurysm was incidentally detected (shown by an arrow), and loss of the olfactory bulb was observed (shown by a circle).

**Figure 2:**
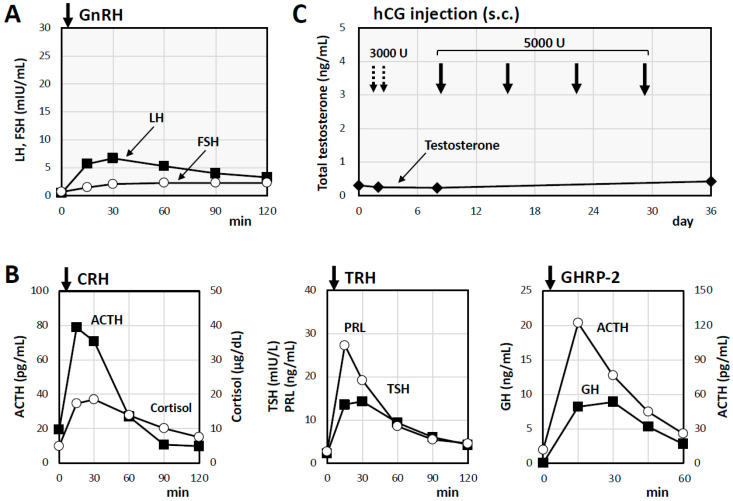
Results of endocrinological tests for the patient with a novel variant of *FGFR1*. (**A**) GnRH test: The GnRH test showed impaired LH and FSH secretion responses. (**B**) CRH, TRH, and GHRP-2 tests: ACTH and cortisol responses and those of PRL and TSH to the CRH test and TRH test, respectively, were almost normal. The GHRP-2 test showed an impaired GH response (maximum GH level of 8.89 ng/mL, 30 min) and a normal ACTH secretion response. (**C**) HCG tests: 3000 units of hCG were subcutaneously administered for 2 consecutive days, followed by weekly administrations of 5000 units of hCG for 4 weeks, resulting in insufficient T responses (maximum T level of 0.43 ng/mL).

A 48-year-old man, who had received surgery for cryptorchidism and who had a history of compression fracture of the second lumbar vertebra and hypertension, visited a department of orthopedics with a chief complaint of lower back pain after a fall from his bed. Computed tomography (CT) examination revealed a new compression fracture of the fourth lumbar vertebra, and bone mineral densities of the lumbar spine and femoral neck had markedly decreased to 62% of the young adult mean (YAM; T-score, −2.8; Z-score, −2.8) and 52% of the YAM (T-score, −3.7; Z-score, −3.2), respectively. Internal medical evaluation of osteoporosis revealed low T levels, and he was referred to our general internal medicine department. He is obese with a body mass index (BMI) of 36.2 kg/m^2^ and high blood pressure of 157/111 mmHg. He exhibited gynecomastia, loss of axillary and pubic hair, and testicular atrophy (Figure 1A). Of note, diminished olfactory function was suspected, and olfactory examination via the Alinamin test and an olfactory threshold test (T&T olfactometry) confirmed anosmia. Laboratory examination revealed elevated liver enzymes, dyslipidemia, and impaired glucose tolerance (Table 1). Abdominal ultrasonography revealed findings consistent with non-alcoholic fatty liver disease (Figure 1B), and spinal CT showed compression fractures of the second and fourth lumbar vertebrae (Figure 1C). Hormonal evaluation revealed anterior pituitary dysfunction and secondary hypogonadism (Table 1). Head MRI revealed no abnormalities in the hypothalamus or pituitary gland, but a loss of the olfactory bulb was observed (Figure 1D). An internal carotid artery aneurysm was incidentally detected, and it was scheduled for elective treatment (Figure 1D). Anterior pituitary function tests were then performed. The tests showed that LH and FSH secretion had low reactions in response to GnRH (up to 6.7 mIU/mL and 2.3 mIU/mL, respectively) (Figure 2A), although the responses to corticotropin-releasing hormone (CRH) and thyrotropin-releasing hormone (TRH) were within the normal range (maximum adrenocorticotropin hormone (ACTH) level, 78.8 pg/mL; maximum cortisol level, 18.5 μg/dL; maximum thyroid-stimulating hormone (TSH) level, 14.3 μIU/mL; and maximum prolactin (PRL) level, 27.3 ng/mL) (Figure 2B). Impaired secretion of growth hormone (GH), but not that of ACTH, in response to GH-releasing peptide-2 (GHRP-2) was also detected (maximum GH level of 8.89 ng/mL < 9 ng/mL) (Figure 2B) [9]. To further evaluate hypogonadism, a human chorionic gonadotropin (hCG) stimulation test was performed. Administration of 3000 units of hCG for two consecutive days followed by weekly administrations of 5000 units for 4 weeks resulted in insufficient T responses (maximum T level of 0.43 ng/mL) (Figure 2C). Considering these results, Kallmann syndrome was clinically diagnosed, genetic testing was performed under genetic counseling by the clinical geneticists of our hospital, and the patient’s written informed consent was obtained. Genetic testing for disorders of sexual maturation (conducted at Kazusa DNA Res. Inst., Kisarazu, Japan) revealed a novel variant (c.2197_2199dupATG, p.Met733dup) in the tyrosine kinase domain of *FGFR1* (also known as *KAL2*), which had not been previously reported in the Human Gene Mutation Database (HGMD^®^), the Genome Aggregation Database (gnomAD), or ClinVar.

In this case, the pathogenicity of the novel variant of *FGFR1* was unclear. Moreover, the results of the GnRH and hCG tests (Figure 2A,C) suggested that the pathophysiology of hypogonadism involved both primary and central factors. Based on these findings, it was hypothesized that this novel variant could affect not only GnRH neurons but also gonadal steroidogenesis, potentially leading to hypogonadism. To investigate this hypothesis, an in vitro study was conducted by using gonadal cells.

## 3. Materials and Methods

### 3.1. Experimental Reagents

Human recombinant basic fibroblast growth factor (bFGF, also known as FGF-2) was purchased from R&D Systems Inc. (Minneapolis, MN, USA). Human granulosa KGN cells were cultured in Dulbecco’s Modified Eagle’s Medium/Ham’s F-12 medium (DMEM/F12) containing 10% fetal bovine serum (FBS) and penicillin–streptomycin (Thermo Fisher Scientific, Waltham, MA, USA) at 37 °C in 5% CO_2_.

### 3.2. Quantitative Real-Time PCR Analysis

KGN cells (1 × 10^5^ cells/mL) were treated with FGF-2 (30 ng/mL) in serum-free DMEM/F12 in 12-well plates for 6 h. Total RNAs were extracted by using TRI Reagent^®^ (Cosmo Bio Co., Ltd., Tokyo, Japan), and RNA concentrations were measured by a NanoDrop^TM^ One spectrophotometer (Thermo Fisher Scientific). The extracted RNA (1 μg) was reverse-transcribed by using ReverTra Ace^®^ (TOYOBO CO., Ltd., Osaka, Japan), and quantitative real-time PCR analysis was performed by using the LightCycler^®^ 96 system (Roche Diagnostic Co., Tokyo, Japan). Primer pairs for ribosomal protein L19 (RPL19), a housekeeping gene; steroidogenic acute regulatory protein (StAR); steroid side-chain cleavage enzyme (P450scc); 3β-hydroxysteroid dehydrogenase 2 (HSD3B2); and aromatase (P450arom) were utilized as we previously reported [10,11]. Other primer pairs used in the present experiment were prepared as follows: fibroblast growth factor receptor 1 (FGFR1), 2584–2603 and 2785–2804 (from GenBank accession #NM_023110.3); sprouty receptor tyrosine kinase (RTK) signaling antagonist 2 (SPRY2), 849–868 and 1047–1066 (NM_005842.4). The primer sets were created from different exons in order to eliminate PCR products derived from chromosome DNA. The relative mRNA levels of the target genes were determined via the Δ threshold cycle (Ct) method. The ΔCt value was calculated by subtracting the Ct value of RPL19 from that of the target genes. Each target gene mRNA level was normalized by the RPL19 mRNA level and expressed as 2^−(ΔΔCt)^.

### 3.3. Transient Transfection of Plasmid Vectors

Plasmid vectors were used to overexpress wild-type FGFR1 or to express mutant FGFR1 found in the patient (c.2197_2199dup, p.Met733dup). The plasmid structures are as follows: the plasmid encoding the wild-type FGFR1, CAG>Kozak>hFGFR1 (NM_023110.3)>SV40 late pA>pUC ori>Ampicillin, and the plasmid encoding the mutant FGFR1, CAG>Kozak>hFGFR1*2197-2199dup>SV40 late pA>pUC ori>Ampicillin, in addition to an empty vector, CAG>Kozak>open reading frame (ORF) stuffer>SV40 late pA>pUC ori>Ampicillin, which are commercially available from VectorBuilder (Kanagawa, Japan). The vector IDs are VB231219-1134eta, VB231219-1382qef, and VB231220-1879eqv, respectively. KGN cells (1 × 10^5^ cells/mL) were cultured, and 1 μg of the plasmid DNA was transiently transfected via Lipofectamine^TM^ 3000 (L3000015, Thermo Fisher Scientific) following the Lipofectamine^TM^ 3000 reagent protocol. After 24 h of culturing, the cells were treated with FGF-2 (30 ng/mL) for 6 h. The medium was then removed, and total RNAs were extracted and subjected to qPCR of the target genes: SPRY2, StAR, HSD3B2, P450scc, and P450arom.

### 3.4. Statistics

All data were obtained from at least three independent experiments performed with triplicate samples and shown as means ± standard error of the mean (SEM). Statistical analysis was performed by using the unpaired *t*-test. *p*-values less than 0.05 were accepted as statistically significant.

## 4. Results

**Figure 3:**
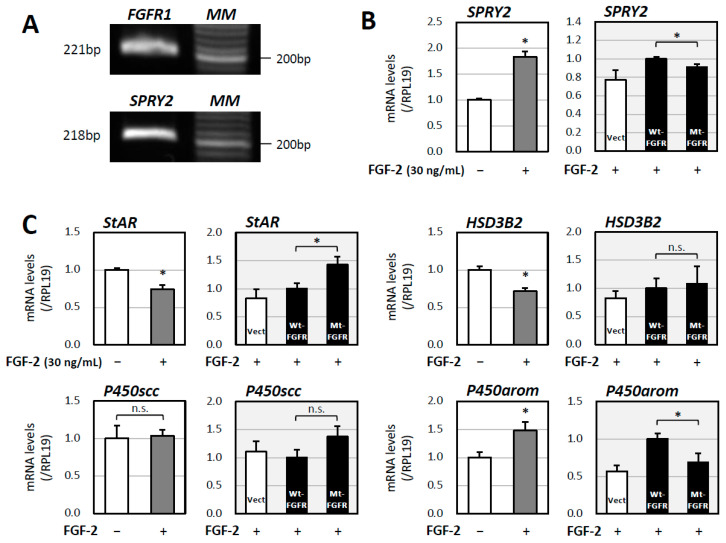
Effects of the novel variant of *FGFR1* on FGFR signaling and steroidogenesis in vitro. (**A**) The expression of mRNAs encoding FGFR1 and SPRY2 was examined via RT-PCR in steroidogenic human granulosa KGN cells. (**B**,**C**) Left panels: KGN cells (1 × 10^5^ cells/mL) were treated with FGF-2 (30 ng/mL) in serum-free DMEM/F12 for 6 h. Total RNAs were extracted, and the mRNA levels of SPRY2, StAR, HSD3B2, P450scc, and P450arom were standardized by RPL19 levels and expressed as fold changes. Results are shown as means ± SEM and were analyzed using an unpaired *t*-test; * *p* < 0.05 vs. basal groups. (**B**,**C**) Right panels: KGN cells (1 × 10^5^ cells/mL) were transfected with 1 μg of an empty vector (Vect) or an expression plasmid encoding wild-type *FGFR1* (Wt-FGFR) or the mutant *FGFR1* (Mt-FGFR) in serum-free DMEM/F12 for 24 h. After FGF-2 (30 ng/mL) stimulation for 6 h, total RNAs were extracted, and the mRNA levels of SPRY2, StAR, HSD3B2, P450scc, and P450arom were standardized by RPL19 levels and expressed as fold changes. Results are shown as means ± SEM and were analyzed using an unpaired *t*-test; * *p* < 0.05 between the indicated groups.

In order to investigate the effects of the novel variant of *FGFR1* found in the patient on gonadal cells, we utilized KGN cells, which are human ovarian granulosa-like tumor cells that are widely used for examining the regulation of gonadal steroidogenesis [12]. It was confirmed that *FGFR1* was expressed in human KGN cells (Figure 3A). We then attempted to evaluate the downstream signaling of FGFR1. FGFRs are expressed on the cell membrane and activated by extracellular signals, in which FGFs play roles as native ligands. Basic FGF (also known as FGF-2) is a prominent member of the FGF family and exerts physiological functions mainly by binding to FGFRs, mainly FGFR1 [13].

The extracellular binding of FGF-2 to FGFR1 activates several intracellular signaling pathways, including mitogen-activated protein kinase (MAPK)/extracellular signal-regulated kinase (ERK), phospholipase C gamma (PLCγ), and phosphatidylinositol-3-kinase (PI3K)/protein kinase B (PKB, also known as Akt) [14]. MAPK can also activate negative regulators of FGF signaling such as sprouty (SPRY) [15]. In the present experiment, SPRY2 was focused on as one of the markers of FGFR1 signaling. We confirmed the expression of SPRY2 in KGN cells (Figure 3A), which was consistent with the results of transcriptomic analysis in a previous study that showed the mRNA expression of SPRY2 in KGN cells [16]. It was revealed that the 6 h FGF-2 (30 ng/mL) treatment upregulated the mRNA levels of SPRY2 by 1.8 fold (Figure 3B), suggesting that SPRY2 can be utilized as a marker for the activation of FGFR1 downstream signaling.

Next, the effects of the novel variant of *FGFR1* on its downstream signaling were evaluated. The plasmid encoding the wild-type *FGFR1* or the plasmid encoding the mutant *FGFR1* (c.2197_2199dup, p.Met733dup) was transfected into KGN cells in the presence of FGF-2. It was demonstrated that the mutant *FGFR1* significantly downregulated the mRNA level of SPRY2 by 9% compared with that in the case of the forced expression of wild-type *FGFR1* (Figure 3B).

Finally, the influence of the novel *FGFR1* variant on gonadal steroidogenesis was investigated. Among gonadal steroidogenic enzymes, the FGF-2 treatment of KGN cells reduced the mRNA levels of StAR and HSD3B2 and increased the mRNA levels of P450arom, while it did not affect the mRNA levels of P450scc (Figure 3C). As shown in Figure 3C, in the presence of FGF-2, transfection with mutant *FGFR1* resulted in the elevation of StAR mRNA levels and a decrease in P450arom mRNA levels compared to that with the forced expression of wild-type *FGFR1*, while the mRNA levels of HSD3B2 and P450scc remained unchanged.

## 5. Discussion

**Figure 4:**
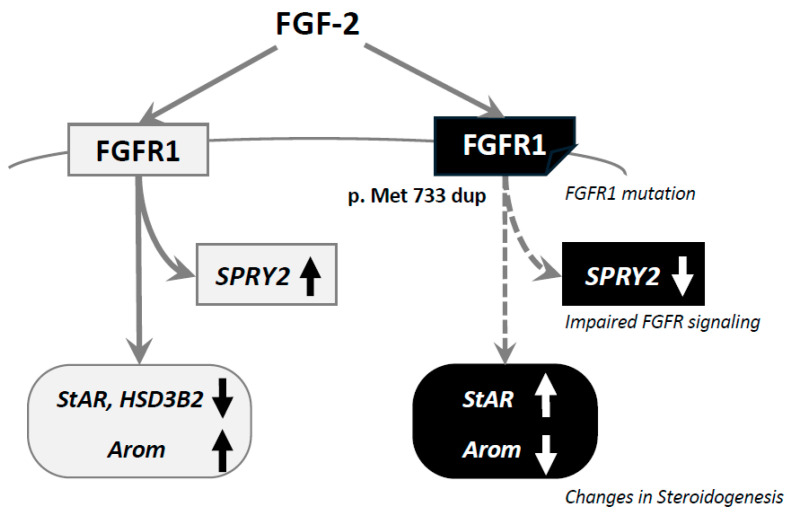
Functions of the novel variant of *FGFR1* in human granulosa cells. FGF-2 stimulates FGFR1 downstream signaling, detected based on an increase in SPRY2 mRNA levels, and affects steroidogenic enzymes by decreasing StAR and HSD3B2 expression and by increasing P450arom expression. The novel variant of *FGFR1* (p.Met733dup) impairs FGFR1 downstream signaling, demonstrated by the downregulation of SPRY2, and changes the expression pattern of steroidogenic enzymes by increasing StAR expression and by decreasing P450arom expression in granulosa cells.

In the present study, the effects of the novel variant of *FGFR1* found in the patient (c.2197_2199dup, p.Met733dup) were investigated using human granulosa KGN cells. It was revealed that the variant impairs FGFR1 signaling, detected through the downregulation of SPRY2, compared with that in the case of the overexpression of wild-type *FGFR1*, which indicates that the variant of FGFR1 affects FGFR1 downstream signaling. It was also found that the variant affects the expression of steroidogenic enzymes of the gonad (Figure 4), indicating that this variant could affect steroidogenesis in gonadal cells in addition to the function of GnRH neurons. Based on the present results, we considered this variant a pathogenic variant [17].

In addition to the present in vitro study, we evaluated the novel variant by using the web-based software MutationTaster2021, which evaluates the pathogenic potential of DNA sequence alterations [18]. The in silico prediction was that the variant is “deleterious”. The methionine residue at 733, duplicated in the variant, comprises the tyrosine kinase domain of FGFR1, which is the most frequent site of *FGFR1* variants found in IHH with a frequency of 41% [2,19]. The residue is conserved across various species, including humans, chimpanzees, macaques, cats, mice, birds, pufferfish, zebrafish, and Drosophila, and is structurally conserved in human FGFR1, FGFR2, FGFR3, and FGFR4 [20], indicating that the residue is pivotal for the physiological function of the FGFR1 protein.

In the present case, the patient showed signs of hypogonadotropic hypogonadism, anosmia, and cryptorchidism but showed no other associated symptoms that are often seen in patients with KS. Recently, from the viewpoint of the genotype–phenotype relationship, it was reported that the presence of multiple gene defects, oligogenicity, results in a more severe KS phenotype [8]. The patient had a single-gene variant and, possibly as a consequence, exhibited relatively uncomplicated clinical features of KS. In these phenotypes detected in KS, the symptoms and signs could be overlooked, potentially resulting in a delayed diagnosis.

In the present study, gonadal cells were used to evaluate the pathogenicity of the novel variant of *FGFR1* since gonadal sex steroid secretion was found to be impaired in an hCG test (Figure 2C). It has been reported that hCG tests reveal poor or subnormal T responses in patients with KS, which could be due to secondary testicular dysfunction resulting from hypogonadotropic hypogonadism [3]. It was reported that peak T levels in 18 patients with KS during the intramuscular administration of 3000 units of hCG for 3 consecutive days ranged from 0.06 to 1.6 ng/mL with a median value of 0.43 ng/mL [3], and those levels were lower than those in a healthy population [21]. In our patient, the T level after the injection of 3000 units of hCG for two consecutive days was 0.26 ng/mL (Figure 2C), which is clearly lower than that in other patients with KS. According to the results of the hCG tests, it was hypothesized that the patient with the novel variant of *FGFR1* (c.2197_2199dup, p.Met733dup) could have an aberrant gonadal steroidogenesis function, which could possess the same variant of *FGFR1* as a germline variant, in addition to the secondary dysfunction resulting from hypogonadotropic hypogonadism typically shown in KS.

In the present case, GH deficiency was also detected by a GHRP-2 test in addition to hypogonadotropic hypogonadism. It has been reported that obesity is associated with lower GH levels and GH responses in GH-provocative tests [22,23]. Since our patient was obese with a BMI of 36.2 kg/m^2^, the interpretation of GH deficiency needs to be approached cautiously. On the other hand, the possibility of combined pituitary hormone deficiency (CPHD) should be considered since heterozygous *FGFR1* variants are reported in 2.7% of cases of CPHD [24]. Since hypogonadotropic hypogonadism and GH deficiency are comorbid in pathogenic *FGFR1* variants [25], it is necessary to carefully monitor the possibility of KS and GHD comorbidity.

There have been some reports showing the pathogenicity of previously unreported variants of *FGFR1* in KS. For instance, Uchida et al. identified a novel pathogenic variant of *FGFR1* (c.1591G>A, p.Glu531Lys) in a patient with KS and holoprosencephaly by using in silico analyses and a three-dimensional model [26]. Gonçalves et al. revealed novel *FGFR1* variants in KS and normosmic idiopathic hypogonadotropic hypogonadism using genetic studies and in silico analyses [27]. As for IHH cases, some studies have confirmed the pathogenicity of *FGFR1* variants by evaluating effects on cell surface receptor levels, transcription reporter activity, kinase activity, and protein expression levels [28,29]. However, there has been no research focusing on the pathogenicity of a novel variant of *FGFR1* in KS with an in vitro study for the direct analysis of the impairment of the downstream signaling of FGFR1. In the present study, the pathogenicity of a novel variant of *FGFR1* was evaluated by an in vitro study, in which human gonadal cells were used to simultaneously investigate the effects on gonadal steroidogenesis.

There has been accumulating evidence indicating that FGF-2 acts on granulosa cells and affects gonadal steroidogenesis [30]. In bovine granulosa cells, FGF-2 inhibits FSH-induced progesterone (P4) and estradiol (E2) production [31]. In rat ovarian granulosa cells, FGF-2 enhances P4 synthesis via the upregulation of StAR mRNA expression [32]. Moreover, FGF-2 enhances FSH-induced P4 synthesis and inhibits FSH-induced E2 synthesis [33]. We have also reported that a related molecule, FGF-8, is involved in the modulation of steroidogenesis, particularly the suppression of E2 production, by cooperating with bone morphogenetic protein (BMP) molecules and activating FSH-induced cAMP-protein kinase A (PKA) signaling via ERK and stress-activated protein kinase/c-Jun NH_2_-terminal kinase (SAPK/JNK) in rat granulosa cells [34,35]. On the other hand, in a study using human ovarian follicles, it was demonstrated that the secretion of E2 was inhibited by the neutralizing antibody against FGF-2 with or without FGF-2 (100 ng/mL) [36], indicating that endogenous FGF-2 has a role in increasing E2 levels. These results suggest that the effects of FGF-2 on follicular steroidogenesis depend on the species and cell types. In our study using KGN cells, it was revealed that FGF-2 (30 ng/mL) suppressed the expression of StAR and HSD3B2 and enhanced the expression of P450arom (Figure 3C), suggesting that FGF-2 may suppress P4 production and enhance E2 production. These results are partially consistent with results obtained by using human ovarian follicles [36]. Moreover, the variant of *FGFR1* changed the expression pattern of these steroidogenic enzymes, suggesting that the novel variant affects gonadal steroidogenesis.

There are some limitations in the present study. First, we did not quantify gonadal steroidogenesis, and the impact of the variant of *FGFR1* on steroid production, therefore, remains speculative. Second, we did not directly compare the expression levels of steroidogenic enzymes in the presence or absence of FGF-2 between each transfected condition. Third, we did not examine the intracellular signaling downstream of FGFR1 that affects steroidogenesis in the present study. Fourth, we used ovarian granulosa cells due to a lack of adequate systems for evaluating steroidogenic capacity in testicular cell lines. Moreover, the effects of the variant of *FGFR1* on GnRH neurons should be investigated with in vitro or in vivo studies in the future.

In conclusion, we reported a case of KS discovered from signs and symptoms of hypogonadotropic hypogonadism and anosmia with a novel variant of *FGFR1* (c.2197_2199dup, p.Met733dup). We then added an in vitro experiment on the function of the variant, and it was revealed that it impairs the functions of FGFR1 and affects steroidogenesis in gonadal cells. The results may provide new insights into the molecular mechanism by which the variant of *FGFR1* impairs gonadal steroidogenesis, in addition to authentic hypogonadotropic hypogonadism, due to the impairment of GnRH neurons, leading to primary, as well as secondary, hypogonadism. This implication may further indicate that we need to change the therapeutic strategy for the hypogonadism of KS based on the responsiveness of steroidogenesis to gonadotropins. Further studies are required to investigate the pathogenesis of the variant in the testes and GnRH neurons.

## Figures and Tables

**Table 1:** Laboratory data on admission.

Analyte	Result	Unit	Reference Interval
Complete blood count			
White blood cells	7980	/μL	[3300–8600]
Red blood cells	442 × 10^4^	/μL	[435 × 10^4^–555 × 10^4^]
Platelets	336 × 10^3^	/μL	[158 × 10^3^–348 × 10^3^]
Biochemistry			
Albumin	3.7	g/dL	[4.1–5.1]
Aspartate transaminase	34	U/L	[13–30]
Alanine transaminase	57	U/L	[10–42]
Alkaline phosphatase	81	U/L	[38–113]
γ-glutamyl transpeptidase	38	U/L	[13–64]
Lactate dehydrogenase	180	U/L	[124–222]
Sodium	137	mmol/L	[138–145]
Potassium	4.1	mmol/L	[3.6–4.8]
Chloride	102	mmol/L	[101–108]
Blood urea nitrogen	10.4	mg/dL	[8–20]
Creatinine	0.57	mg/dL	[0.65–1.07]
Triglyceride	108	mg/dL	[40–234]
Low-density lipoprotein cholesterol	154	mg/dL	[65–163]
Fasting plasma glucose	119	mg/dL	[73–109]
Hemoglobin A1c	6.2	%	[4.9–6.0]
Endocrine data			
Cortisol	6.9	μg/dL	[7.1–19.6]
Adrenocorticotropin	20.8	pg/mL	[7.2–63.3]
Free thyroxine	1.47	ng/dL	[0.97–1.69]
Thyroid-stimulating hormone	1.81	mIU/L	[0.61–4.23]
Prolactin	3.5	ng/mL	[4.3–13.7]
Growth hormone	0.04	ng/mL	[0–2.47]
Insulin-like growth factor -I	57.8	ng/mL	[89–248]
Follicle-stimulating hormone	0.7	mIU/mL	[1.8–12]
Luteinizing hormone	0.4	mIU/mL	[2.2–8.4]
Testosterone	0.29	ng/mL	[1.87–9.02]
Free testosterone	1.4	pg/mL	[4.7–21.6]
Vasopressin	0.9	pg/mL	[0–2.8]
Osmolality	281	mOsm/kg	[276–292]

## Data Availability

The original contributions presented in this study are included in the article. Further inquiries can be directed to the corresponding author.
